# Redundancy Among Parameters Describing the Input-Output Relation of Motor Evoked Potentials in Healthy Subjects and Stroke Patients

**DOI:** 10.3389/fneur.2019.00535

**Published:** 2019-05-21

**Authors:** Claire Kemlin, Eric Moulton, Sara Leder, Marion Houot, Sabine Meunier, Charlotte Rosso, Jean-Charles Lamy

**Affiliations:** ^1^Institut du Cerveau et de la Moelle épinière, ICM, Inserm U 1127, CNRS UMR 7225, Sorbonne Université, Paris, France; ^2^APHP, Urgences Cérébro-Vasculaires, Hôpital de la Pitié Salpêtrière, Paris, France; ^3^AP-HP, Department of Neurology, Hôpital de la Pitié-Salpêtrière, Centre of excellence of neurodegenerative disease (CoEN), Institute of Memory and Alzheimer's Disease (IM2A), ICM, CIC Neurosciences, Paris, France

**Keywords:** transcranial magnetic stimulation, input-output curve, corticospinal excitability, motor evoked potentials, stroke

## Abstract

**Background:** Transcranial magnetic stimulation (TMS) is widely used to probe corticospinal excitability through Motor Evoked Potential (MEP) amplitude measurements. The input-output (I/O) curve is a sigmoid-shaped relation between the MEP amplitude at incremented TMS intensities. The aim of this study was to examine the relationships between seven parameters derived from the sigmoid function.

**Methods:** Principal Component Analysis and Spearman's rank correlation matrices were used to determine if the seven I/O curve parameters capture similar or, conversely, different aspects of the corticospinal excitability in 24 healthy subjects and 40 stroke survivors with a hand motor impairment.

**Results:** Maximum amplitude (MEP_max_), peak slope, area under the I/O curve (AUC), and MEP amplitude recorded at 140% of the resting motor threshold showed strong linear relationships with each other (ρ > 0.72, *p* < 0.001). Results were found to be similar in healthy subjects and in both hemispheres of stroke patients. Our results did not support an added benefit of sampling entire I/O curves in both healthy subjects and stroke patients, with the exception of S_50_, the stimulus intensity needed to obtain half of MEP_max_ amplitude.

**Conclusions:** This demonstrates that MEP elicited at a single stimulus intensity allows to capture the same characteristics of the corticospinal excitability as measured by the AUC, MEP_max_ and the peak slope, which may be of interest in both clinical and research settings. However, it is still necessary to plot I/O curves if an effect or a difference is expected at S_50_.

## Introduction

Transcranial magnetic stimulation (TMS) is widely used to probe corticospinal excitability in both healthy subjects and in a broad range of neuropsychiatric conditions. A common approach from basic research to pivotal clinical trials is to compare recruitment curves of TMS-induced motor evoked potentials (MEPs) between groups of subjects or before and after different types of interventions aimed at promoting brain plasticity (i.e., pharmacotherapy or non-invasive brain stimulation).

**Figure 1:**
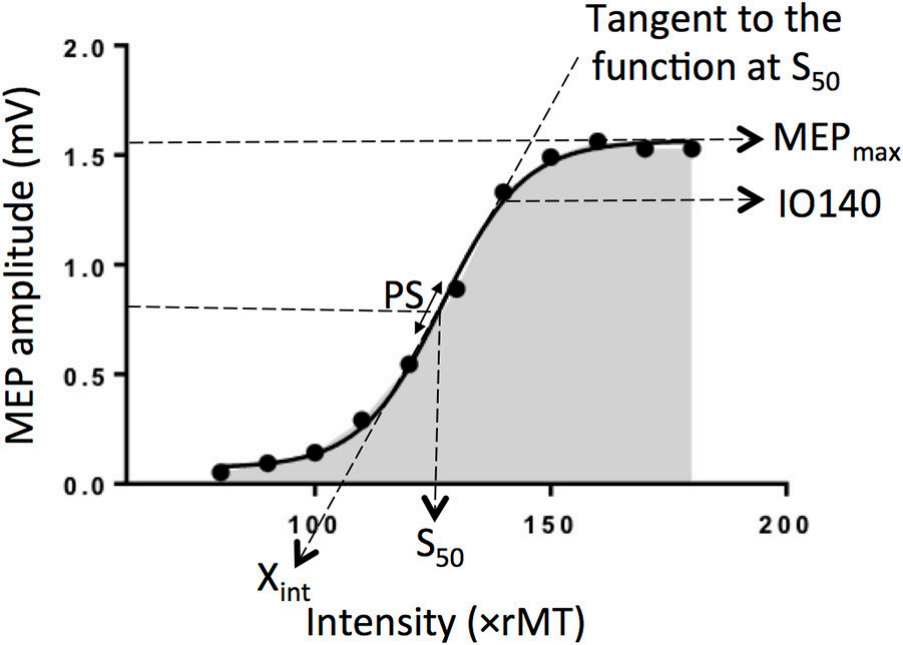
Electrophysiological parameters extracted from an example of an input output curve (I/O curve) fitted by a sigmoid function. Are shown the following variables: X_int_: X intercept, IO140: Motor Evoked Potential amplitude recorded at 140% rMT, PS, peak slope; AUC, area under the I/O curve in gray; S_50_, stimulus intensity needed to obtain 50% of the maximum response; MEP_max_, maximum value of the sigmoid function.

The input-output (I/O) relation in the corticospinal pathway is assessed by plotting MEP amplitude *vs*. stimulus intensity and fitting the data with the following sigmoid function equation (1–4): $MEP\left(s\right)=MEP_{\max}/\left(1+\exp^{m\left(S_{50}-s\right)}\right)$, where *MEP*(*s)* is the MEP amplitude at the stimulation intensity *s*, MEP_max_ is the maximum MEP amplitude, S_50_ is the stimulus intensity needed to obtain 50% of MEP_max_ amplitude, and m is the slope parameter of the sigmoid function, i.e., the global slope of the function (Figure 1). Three additional parameters can be derived from the I/O curve: (1) the peak slope (PS), i.e., the instantaneous slope of the ascending limb of the curve at S_50_, which reflects the recruitment gain of motoneurons and is given by the formula: *PS* = *m* **x** *MEP*_max_/4, (2) the x-intercept (X_int_) of the tangent at S_50_, and (3) the area under the I/O curve (AUC) usually calculated using the trapezoidal area method (5).

To date, the inter-dependency between all these parameters are not fully understood. Indeed, although the PS depends on both m parameter and MEP_max_, it does not mean these three parameters are correlated together. Same for X_int_, which depends on m parameter and S_50_. The question arises whether these variables capture similar or, conversely, different aspects of the corticospinal excitability and if so, how each of them relates to one other. To clarify the interdependency between these parameters, we estimated I/O curves from the dominant hemisphere of healthy volunteers and performed Principal Component Analyses (PCA) in addition to correlation matrices to summarize the most important linear relationships between variables. PCA is a tool capable of summarizing the most important linear relationships between variables and computing synthetic variables from the original variables named principal components (PCs). PCA provides a visual and geometric representation of the correlation matrix (6, 7). In a second step, to test whether our results could be extrapolated to patients suffering from neurological conditions, we performed the same analyses on data collected in both the affected and unaffected hemispheres of stroke patients given that this population represents the most frequent brain damaged disease worldwide. Indeed, the sigmoid function has been previously shown to be a reliable method to plot IO curve in stroke patients (4).

## Materials and Methods

### Subjects

Data were extracted from a parallel study (Core protocol: NCT 02284087) and were collected before the intervention stage of the study. The study was approved by the appropriate legal and ethical authority (CPP Ile de France VI—Pitié-Salpêtrière) in accordance with the Declaration of Helsinki. Written informed consent was obtained from all participants.

Inclusion criteria for healthy participants were (1) no history of neurological or psychiatric disorders, (2) Mini-Mental State Examination ≥27, (3) age older than 18 years, (4) no contraindications to TMS and, (5) no use of psychoactive medication or recreational drugs.

Patients were recruited according the following inclusion criteria: (1) ischemic stroke >1 month old, (2) no history of psychiatric disorders as determined through an interview by a trained neurologist, (3) Mini-Mental Status Examination ≥27, (4) age older than 18 years, (5) no contra-indications for TMS, and (6) presence of an upper-limb deficit (Fugl-Meyer assessment scale for upper extremity without reflex activity items <60) with some preserved hand movement (maximum finger flexors voluntary contraction >0 Newton).

All subjects were tested with the Edinburgh handedness inventory (EHI) with a cut-off of 0.2 to be considered as right-handed (8, 9).

### TMS Evaluation

Subjects were comfortably seated. Surface EMGs were recorded from the first dorsal interosseous (FDI) muscles of both hands at rest, in a belly-tendon montage using Ag/AgCl surface electrodes (Kendall, Chicopee, MA). EMG signals were amplified (×1000), filtered with a band pass of 0.02–2 kHz (Digitimer D360, Digitimer Ltd., Hertfordshire, UK), digitalized at 5 kHz via a Power 1401 data acquisition interface (Cambridge Electronics Design, Cambridge, UK) and stored for *offline* analysis with the Signal software (version 5.02a, Cambridge Electronics Design, Cambridge, UK). Anatomical T1-weighted Magnetic Resonance images were entered into a computerized frameless stereotaxic system (Brainsight2, Rogue Research, Inc., Montreal, Canada). MEPs were elicited using a MAGSTIM 200^2^ stimulator (Magstim, Dyfed, UK) connected to a figure-of-eight-shaped coil with an internal wing diameter of 7 cm. The handle of the coil was held pointing postero-laterally in order to induce a current in the brain from the posterior-lateral to the anterior-medial (PA) orientation. The coil was first held tangentially to the scalp over the presumed hand knob area as determined by the anatomical 3D reconstruction of each participant's brain. The optimal coil position was then determined as the site where TMS at a suprathreshold intensity consistently produced the largest MEPs in the contralateral FDI muscle. The coil position was continuously monitored using a neuronavigation system to ensure its constant position over the “hotspot” tangentially to the head surface. TMS pulses were delivered at 0.2 Hz. The resting motor threshold (rMT) was defined in both hemispheres as the minimum stimulation intensity needed to elicit recordable MEPs in the relaxed FDI of >50 μV in 5 out of 10 consecutive trials (10).

To sample the I/O curve, eight MEPs were collected at each stimulus intensity ranging from 80 to 180% rMT (or until the maximum stimulator output was reached) in an incremental order with steps of 10% rMT according to the IFCN guidelines (10). Individual MEP trials were examined *offline* and those showing voluntary EMG activity in the 100 ms prior to stimulus artifact resulting in MEP amplitude facilitation (>2 SD of mean MEP amplitude without EMG) were discarded and peak-to-peak MEP amplitudes were measured in the remaining trials (11).

This procedure was carried out in the dominant hemisphere of healthy subjects and in both the affected and unaffected hemispheres in stroke patients. The following seven electrophysiological parameters were computed *offline* **using GraphPad Prism software (version 6.05)** for subsequent statistical analysis: MEP_max_, S_50_, m, PS, X_int_, AUC and MEP amplitude at 140% rMT derived from the I/O curve (IO140) according to the IFCN recommendations (12). The coefficients of fitting (*R*^2^) of the I/O curve were also calculated.

### Statistical Analysis

To examine the relationships between the seven different electrophysiological variables, Principal Component Analysis (PCA) were performed separately for the dominant hemisphere of the healthy subjects and for each hemisphere of stroke patients with the seven electrophysiological variables (6, 7). Additional PCAs were computed by including MEP amplitude sampled at five other stimulus intensities (110, 120, 130, 150, and 160% rMT) to assess whether results obtained at IO140 could be extrapolated to other intensities.

Because of the non-normal distribution of the data, as assessed by Shapiro-Wilk's tests, Spearman's rank coefficient matrices were used to examine relationships between electrophysiological variables. Confidence intervals were obtained through bootstrapping with 5,000 iterations (13).

Furthermore, we tested the absence/presence of multicollinearity to demonstrate equivalence. To that purpose, the variance inflation factor (VIF) was computed using JASP (Version 0.8.6, Amsterdam, The Netherlands). By convention, multicollinearity is considered present if the VIF of one variable is higher than 10 (14).

Wilcoxon paired signed-rank tests were employed to assess statistical differences in I/O curve parameters between hemispheres in stroke patients.

*P*-values for each correlation matrix were adjusted using the Benjamini-Hochberg method (15). Statistical analyses were performed using R software (Version 3.5.0), package FactoMineR for the PCA (6).

## Results

Twenty-four healthy participants [10 females (41.7%)], median age: (29 years [Q1: 25th percentile -Q3: 75th percentile: 27–31], min–max: 22–65 years) and forty stroke patients [14 females (35%)], median age: (63 years [57–71], min-max: 33–82), median time since stroke: 106 days [48–276], min-max: 31–1,764) with upper limb motor impairment were enrolled in the study.

All subjects but two healthy participants and one patient, were right-handed. Table 1 summarized the characteristics of the patients.

**Table 1:** Characteristics of the 40 patients.

**Demographic characteristics**	
Gender	26 males (65%)
Age (years)	63 [57–71] (33–82)
**Stroke characteristics**	
Time post stroke onset (months)	3.5 [1.6–9.1] (1.02–58.8)
Lesion side	20 left sided stroke (50%)
Lesion localization	24 SC (60%), 16 CSC (40%)
Lesion volume (cm^3^)	5.7 [1.7–20.8] (0.8–209.0)
**Upper limb motor function**	
UE FM	52 [41–55] (5–58)
JTT ratio	2 [1.4–12.8] (0.9–26.4)
mGS ratio	0.5 [0.2–0.8] (0.05–0.9)

*Results are presented as median [Q1–Q3] (min–max) for the continuous variables and as number and percentage for binary variable. Q1 and Q3 represent the 25th and 75th percentile, SC, subcortical; CSC, cortico-subcortical; UE FM, Upper Extremity Fugl-Meyer; JTT, Jebsen Taylor Hand Function test; mGS, maximal grip strength*.

Electrophysiological parameters in the 24 healthy subjects are summarized in Table 2 and in Supplementary Figure 1.

**Table 2:** Electrophysiological parameters in the dominant hemisphere of healthy subjects.

rMT (%MSO)	41 [34–47] (29–56)
X_int_ (%rMT)	110 [102–119] (82–142)
IO140 (mV)	1.34 [0.80–2.70] (0.35-6.93)
MEP_max_ (mV)	3.34 [1.63–5.90] (0.52–9.77)
PS (mV/%rMT)	0.062 [0.033–0.129] (0.010–0.320)
AUC (mVx%rMT)	205.3 [76.42–294.8] (22.7–607.3)
m (mV/%rMT)	0.070 [0.060–0.110] (0.040–0.250)
S_50_ (%rMT)	139 [125–148] (110–170)
*R*^2^	0.98 [0.96–0.98] (0.86–0.99)

*Results are presented as median [Q1–Q3] (min–max). Q1 and Q3 represent the 25th and 75th percentile, rMT, resting motor threshold; MSO, maximal stimulator output; X_int_, X intercept, IO140: Motor Evoked Potentials amplitude recorded at 140% rMT; MEP_max_, maximum value of the sigmoid function; PS, peak slope; AUC, area under the input output curve; m, slope; S_50_, stimulus intensity needed to obtain 50% of the maximum response; R^2^, coefficients of fitting*.

**Figure 2:**
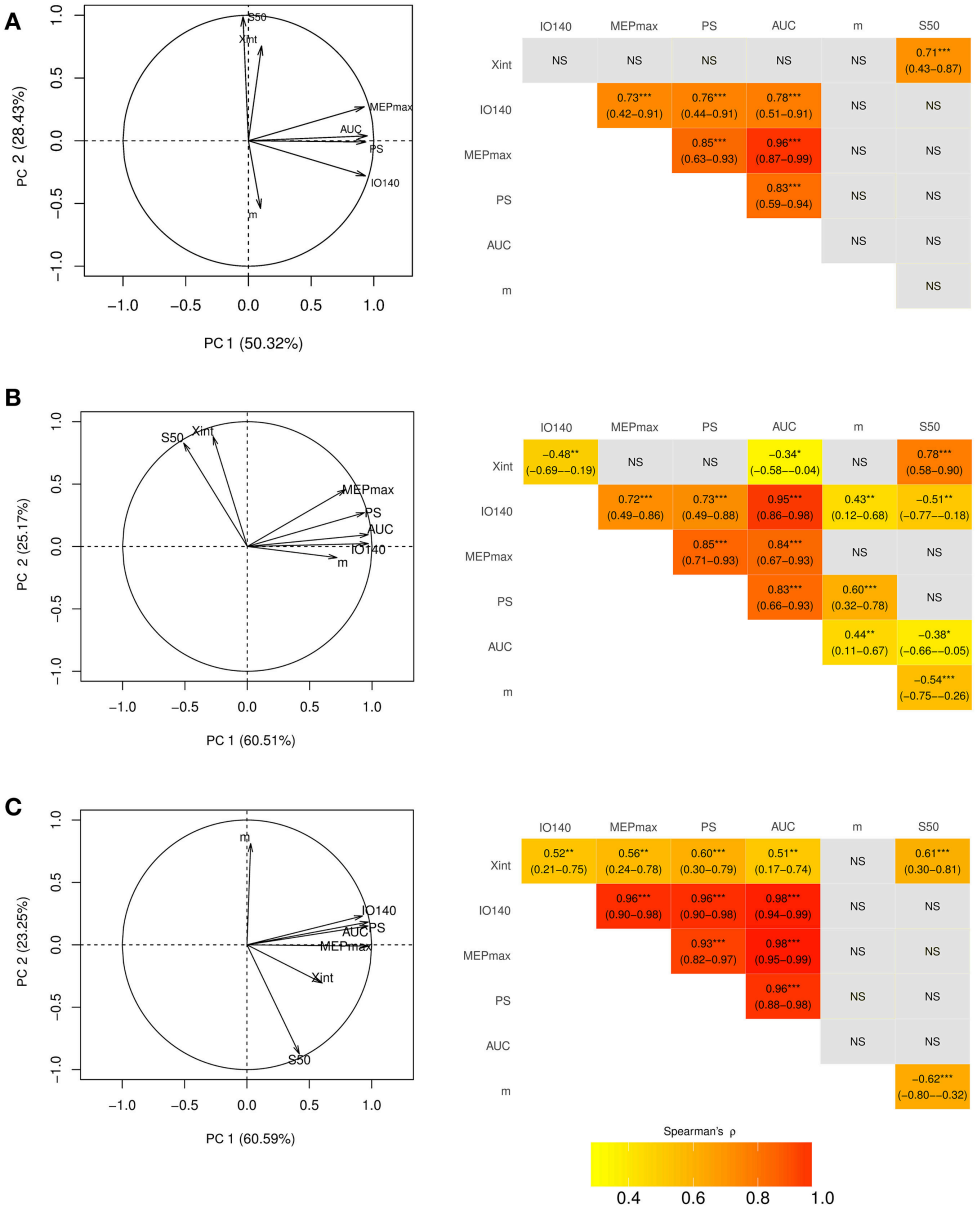
Correlation circle created with the first two components of the principal component analysis (PCA) (left column) and corresponding correlation matrices (right column) in the dominant hemisphere of **(A)** healthy subjects and **(B)** in the unaffected and **(C)** affected hemisphere of stroke patients. On PCA graphs are plotted the seven electrophysiological variables on a plane spanned by PC1 and PC2. In the correlation matrices are presented the spearman correlation coefficients with their 95% Confidence Interval. X_int_, X intercept; IO140, Motor Evoked Potential amplitude recorded at 140% rMT; MEP_max_, maximum value of the sigmoid function; PS, peak slope; AUC, area under the input output curve, m, slope; S_50_, stimulus intensity needed to obtain 50% of the maximum response. *** <0.001, ** <0.01, * <0.05, NS, non-significant.

The first two principal components explained 79% of the total variance of the seven electrophysiological variables (Figure 2A) in healthy subjects. MEP_max_, PS, AUC, and IO140 showed strong positive correlations with each other. Figure 2A showed that the arrows of these 4 variables clustered together and the Spearman's rank coefficients in the correlation matrix were above 0.7 indicating high positive correlation (16). Nevertheless, all of these 4 variables were independent from S_50_ since (i) the arrow of the PCA was perpendicular to others and, (ii) the correlations were not significant. Because X_int_ and *m* were not well-explained by the two first principal components, their relation with the other variables could not be interpreted from the correlation circle.

Electrophysiological parameters in 40 stroke patients are summarized in Table 3 and in Supplementary Figure 1. MEPs could not be elicited in the affected hemisphere of five patients (12%). As expected, all electrophysiological parameters strongly differed between the unaffected and the affected hemispheres (Table 3).

**Table 3:** Electrophysiological parameters in stroke patients.

	**Unaffected Hemisphere**	**Affected Hemisphere**	**Adjusted *p*-value ^**Δ**^**
rMT (%MSO)	42 [38–48] (26–65)	50 [42–60] (29–75)	^**^
X_int_ (%rMT)	110 [105–121] (84–146)	104 [91–108] (74–127)	^*^
IO140 (mV)	1.71 [0.90–2.77] (0.2–9.5)	0.32 [0.16–1.63] (0–10.2)	^**^
MEP_max_ (mV)	3.32 [1.80–5.03] (0.3–11.3)	0.43 [0.19–2.48] (0–10.7)	^***^
PS (mV/%rMT)	0.066 [0.035–0.124] (0.010–0.438)	0.014 [0.048–0.076] (0.001–0.383)	^***^
AUC (mVx%rMT)	142.20 [77.54–204.40] (18.2–630.9)	33.57 [13.07–138.50] (0–571)	^***^
m (mV/%rMT)	0.080 [0.060–0.110] (0.030–1.000)	0.10 [0.07–0.15] (0.040–0.220)	ns
S_50_ (%rMT)	135 [123–150] (111–184)	124 [112–134] (94–154)	^*^
*R*^2^	0.97 [0.95–0.99] (0.71–0.99)	0.96 [0.94–0.98] (0.71–0.99)	ns

*Results are presented as median [Q1–Q3] (min–max). Q1 and Q3 represent the 25th and 75th percentile. Wilcoxon signed-rank tests were computed between the affected and the unaffected hemispheres in 33 patients with recordable MEP in both hemispheres*.

*** p < 0.001,

** p < 0.01,

* *p < 0.05, ns, not significant; MEP, motor evoked potential; rMT, resting motor threshold; MSO, maximal stimulator output; X_int_, X intercept; IO140, Motor Evoked Potentials amplitude recorded at 140% rMT; MEP_max_, maximum value of the sigmoid function; PS, peak slope; AUC, area under the input output curve; m, slope; S_50_, stimulus intensity needed to obtain 50% of the maximum response*.

The first two principal components of the PCA explained 86% of the total variance of the seven electrophysiological variables in the unaffected hemisphere and 84% in the affected hemisphere in stroke patients (Figures 2B,C). Despite striking differences between I/O parameters of the affected and unaffected hemispheres, MEP_max_, PS, AUC, and IO140 strongly intercorrelated, regardless of the hemisphere considered (Figures 2B,C). However, the correlations between these 4 parameters were even stronger when considering the affected hemisphere (ρ > 0.9) than the unaffected one (ρ > 0.7). Moreover, S_50_ was independent from the four correlated variables. Only interpretable in the unaffected hemisphere, X_int_ was highly correlated with S_50_. The slope, m, was not interpretable by the first two principal components in either hemisphere.

In addition, although the time post stroke ranged from 48 to 276 days, there was no correlation between this delay and the I/O parameters for both the affected and unaffected hemispheres. Similarly, the I/O parameters did not differ according to the stroke location (subcortical or cortico-subcortical location).

The variance inflation factor (VIF) is reported for each I/O parameter in the healthy subjects, unaffected and affected hemispheres of the stroke patients in Supplementary Table 1. For each population, the VIF was above 10, indicating multicollinearity for the same four variables as the PCA: IO140, MEP_max_, PS, and AUC. However, it confirmed that the S_50_ was independent and showed also that m and Xint were not highly correlated.

**Figure 3:**
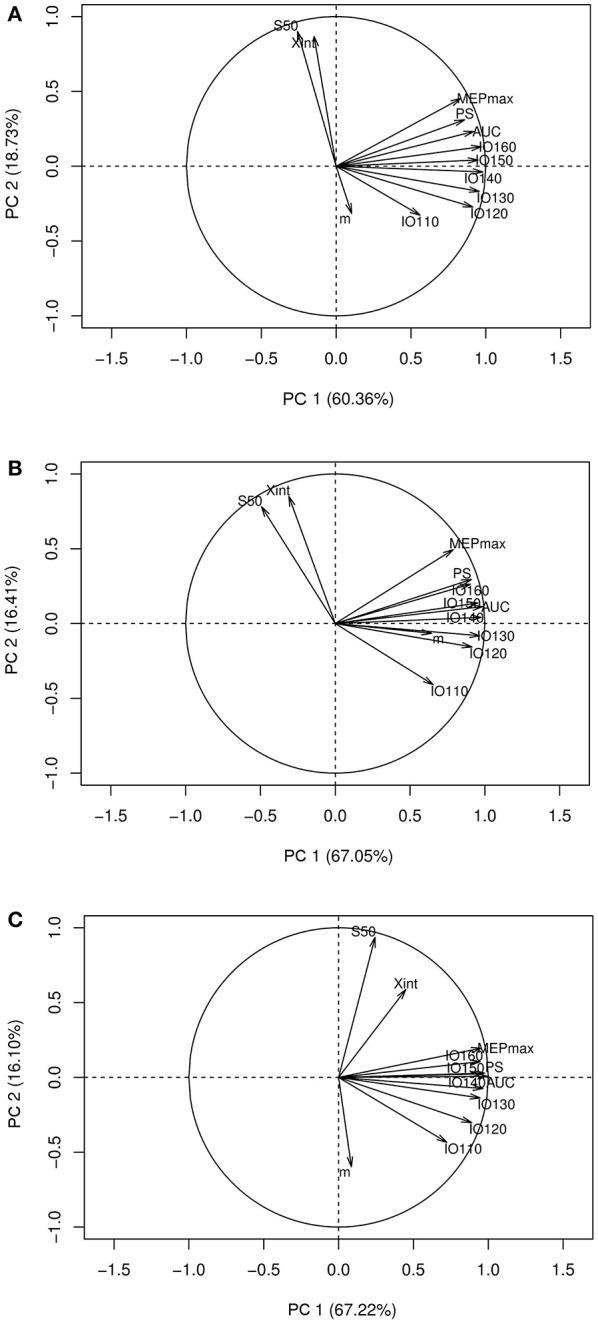
Correlation circles with the I/O curve parameters and the MEP amplitude at stimulation intensities ranging from 110 to 160% rMT. **(A)** PCA graph in healthy subjects, **(B)** PCA graph in stroke patients in the unaffected hemisphere, **(C)** PCA graph in stroke patients in the affected hemisphere. PCA, principal component analysis; X_int_, X intercept, IO110, IO120, IO130, IO140, IO150, IO160: Motor Evoked Potentials amplitude recorded at 110, 120, 130, 140, 150, and 160% rMT, respectively; MEP_max_, maximum value of the sigmoid function; PS, peak slope; AUC, area under the input output curve; m, slope; S_50_, stimulus intensity needed to obtain 50% of the maximum response.

Interestingly, in healthy subjects and stroke patients (unaffected hemisphere), the relationships between the seven electrophysiological variables were similar for MEP amplitudes ranging from 120 to 160% rMT but not when MEP were collected at 110% rMT Figure 3.

## Discussion

There are two new findings in our results. (1) Four I/O curve-derived parameters, namely MEP amplitude recorded at 140% rMT, MEP_max_, PS, and AUC, were clustered in healthy participants independently from S_50_. (2) Despite brain damage, I/O curve-derived parameters were similarly clustered and thus intercorrelated in both hemispheres of stroke patients independently from the time post-stroke or the lesion location.

One could expect that adding parameters from the I/O curve (e.g., MEP_max_, PS, and AUC) to a single measure of MEP amplitude collected at a given intensity (140% rMT) would provide further information when assessing corticospinal excitability. However, this notion is challenged by the high degree of correlation found between MEP_max_, PS and AUC from the I/O curve and MEP amplitude at 140% rMT. Additionally, this relationship exists when MEPs are collected between 120 and 160% rMT; this means that the AUC, MEP_max_, and PS can be dropped when measuring MEPs at those intensities.

It is worth noting that the four correlated variables, in fact, reflect the excitability of the entire corticomotoneuronal pathway, from the cortical cells to the motoneuron pool including spinal interneuronal relays (1, 17). More specifically, MEP_max_ reflects the maximum corticospinal output from the TMS pulse whereas PS represents its gain (18). A good correlation between these two variables was expected since PS is proportional to MEP_max_ (see Introduction). This was also expected for AUC, which is seen as a surrogate marker of the overall corticospinal output and is derived from the gradual summation of outputs as inputs increase. Its value should therefore also strongly relate to the maximum output (i.e., MEP_max_) (5, 19–21).

In stroke patients, despite the striking differences between hemispheres (22), the four aforementioned variables also intercorrelated as in healthy subjects regardless of the hemisphere considered. This suggests that collecting few MEPs at a given stimulus intensity is sufficient to capture the characteristics of the corticospinal excitability measured by the AUC, MEP_max_, and the peak slope after stroke. Moreover, this method of assessing corticospinal excitability requires fewer pulses and is thus by far much faster than sampling the full I/O curve, which may be useful in a clinical setting or in research protocols when accounting for patient fatigue (23) or the short-lived aftereffects of an intervention.

Importantly, even if these four variables are highly intercorrelated, our results do not imply that interventions (i.e., pharmacotherapy, non-invasive brain stimulation, etc…) will affect all of these parameters similarly. For example, it has been shown that low and high frequency rTMS exerts a more complex influence on cortical network excitability, as assessed by an I/O curve, than simple inhibitory and facilitatory effects, usually assessed with MEP amplitudes collected at a single intensity (24). Similarly, GABA_A_ agonists selectively decreases MEP amplitude only in the high-intensity part of the I/O curve (25, 26), suggesting that I/O curves can still be collected in interventional studies. Furthermore, we found that S_50_ is independent from the four other variables. This finding was foreseen since S_50_ is one parameter that reflects the intensity of the TMS output while others relate to amplitude. Indeed, the S_50_ parameter is necessary to induce a MEP amplitude equidistant between resting motor threshold and MEP_max_. This amplitude is commonly used to assess the effect of an intervention on corticospinal excitability or to explore intracortical circuits using paired-pulses paradigms (i.e., short interval intracortical inhibition or intracortical facilitation).

Finally, using a single stimulus intensity does not allow the operator to know how large is the portion of the motoneuronal pool activated. However, as displayed in Figure 3, these relationships were identical with MEPs collected at other stimulation intensities ranging from 120 to 160% rMT (i.e., when late I-waves are recruited) but not at 110% rMT (i.e., when early I-waves are elicited) (27). Since late I-waves but not the I1 wave show different sensitivity to several interventions (27), it may still be useful to collect I/O curve when early I-waves are thought to mediate the effects of a specific intervention.

Considering the short interstimuli interval (5 s), the incremental order of stimulus intensity may induce a hysteresis effect with a systematic rightward shift of the I/O curve (28). However, the effect of the stimulation intensity order is controversial (29). Indeed, we had chosen the incremental order to limit MEP amplitude variability.

Our study has several limitations. First, we did not study the test-retest reliability of each metric of the I/O curve, which can be another deciding factor beyond redundancy to determine which variable to drop. However, in previous studies, the intraclass coefficients of the IO parameters for the FDI muscle has been shown to be good to excellent (>0.6), indicating a good reliability in healthy subjects (3, 30) and in stroke patients (31). How our results obtained in healthy subjects and stroke patients can be extrapolated to other neurological conditions still need to be determined especially in diseases associated with motoneuronal loss such as amyotrophic lateral sclerosis. Second, healthy controls were younger than stroke patients. Nevertheless, the goal of the study was not to compare them but to extrapolate the results from one population to another. Third, we only use the sigmoid function and not other equations for fitting the IO curve, as it has been shown to best fit the IO relationship (1, 32). Indeed, our *R*^2^ values were excellent in both healthy subjects and stroke patients. Finally, given that our parameters were collected at rest only, we cannot extrapolate our observations to data collected under an active condition.

## Conclusions

We examined the relationship between I/O curve parameters and MEP amplitude recorded at a single intensity. Although I/O curve analysis can convey some additional information, our results did not overall support an added benefit of sampling entire recruitment curves in both healthy subjects and stroke patients, except if an effect or a difference is expected at S_50_. However, to what extent our results rely on individual characteristics in neuronal function or anatomy such as the number or the coherence of corticospinal tract fibers is not clear and unknown. Nevertheless, this finding may raise doubts about the pertinence of systematically acquiring such measurements, especially in both clinical and research settings.

## Ethics Statement

The study was approved by the appropriate legal and ethical authority (CPP Ile de France VI—Pitié- Salpêtrière) in accordance with the Declaration of Helsinki. Written informed consent was obtained from all participants.

## Author Contributions

J-CL and CR were involved in the conception and the design of the project. They also participated in the data collection with EM, SL, and CK. CK has analyzed the data with MH and drafted the manuscript with CR and J-CL. SM was involved in the interpretation of the results and has revised it critically for important intellectual content. EM, native English speaker, has also proofread the text.

### Conflict of Interest Statement

The authors declare that the research was conducted in the absence of any commercial or financial relationships that could be construed as a potential conflict of interest.
