# Supplementary material for: Redundancy Among Parameters Describing the Input-Output Relation of Motor Evoked Potentials in Healthy Subjects and Stroke Patients
Source: Front Neurol. 2019 May 21;10:535. doi: 10.3389/fneur.2019.00535 (PMC6537607; doi:10.3389/fneur.2019.00535)
Supplement: Supplementary file 1 [file Data_Sheet_1.docx]

# Supplementary Material

**Supplementary Figure legend:** Averaged I/O curves of healthy volunteers (black line) and of both the unaffected (grey line) and the affected (grey dotted line) hemisphere of stroke patients.


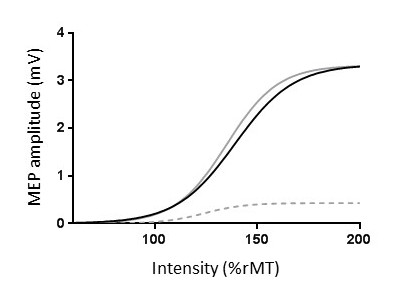


**Supplementary Table 1: Variation inflation factor of the seven electrophysiological parameters:** IO140: Motor Evoked Potential amplitude recorded at 140% rMT, MEP_max_: maximum value of the sigmoid function, PS: peak slope, AUC: area under the I/O curve, S_50_: stimulus intensity needed to obtain 50% of the maximum response, X_int_: X intercept, m: slope of the sigmoid function.

| **Population** | **Healthy subjects** | **Stroke Patients** | |
| --- | --- | --- | --- |
| **Hemisphere** | **Dominant hemisphere** | **Unaffected hemisphere** | **Affected hemisphere** |
| **IO140** | **16** | **27** | **43** |
| **MEP_max_** | **12** | **11** | **62** |
| **PS** | **20** | **14** | **40** |
| **AUC** | **11** | **41** | **139** |
| **S_50_** | **5** | **8** | **8** |
| **X_int_** | **9** | **7** | **6** |
| **m** | **4** | **2** | **5** |
